# Critical Finding of Wellens' Syndrome in a Patient who Presented With a Fibular Fracture

**DOI:** 10.7759/cureus.9652

**Published:** 2020-08-11

**Authors:** Khaled Alabdallah, Sami Rabah, Shazia Aziz, Mohammad Aldiabat, Donya Bani Hani

**Affiliations:** 1 Internal Medicine, Lincoln Medical Center, New York City, USA

**Keywords:** t wave, ecg, coronary artery disease, biphasic t waves, cardiology, lad stenosis, wellens' syndrome

## Abstract

Diagnosing myocardial infarction is not always straightforward; there are many insidious presentations that can be overlooked resulting in deadly consequences. We present a 76-year-old male who came to the ED complaining of right ankle pain. A routine electrocardiogram (ECG) done was noted to have biphasic T waves in leads V2 and V3 which was characteristic of Wellens' syndrome. Subsequent workup showed an increase in troponin T levels in a chest pain-free setting. The patient underwent urgent cardiac catheterization which showed significant triple vessel coronary artery disease, with 90% proximal occlusion of the left anterior descending artery, eventually requiring coronary artery bypass grafting (CABG). Timely diagnosis and management prevented serious consequences of his underlying severe coronary artery disease.

## Introduction

Wellens’ syndrome is a unique pattern of T-wave electrocardiographic (ECG) changes that are associated with critical stenosis of the proximal left anterior descending (LAD) artery [1]. Recognizing this ECG pattern is extremely important as these patients are considered at high risk of developing an extensive anterior wall acute myocardial infarction (MI) [1]. Approximately 75% of patients with Wellens’ syndrome develop acute anterior wall MI within weeks if they were treated medically without surgical intervention [1,2]. Typically, this unique ECG pattern manifests when the patient is pain-free [3]. We hereby describe a symptomless presentation of Wellens’ syndrome with characteristic ECG findings. 

## Case presentation

**Figure 1:**
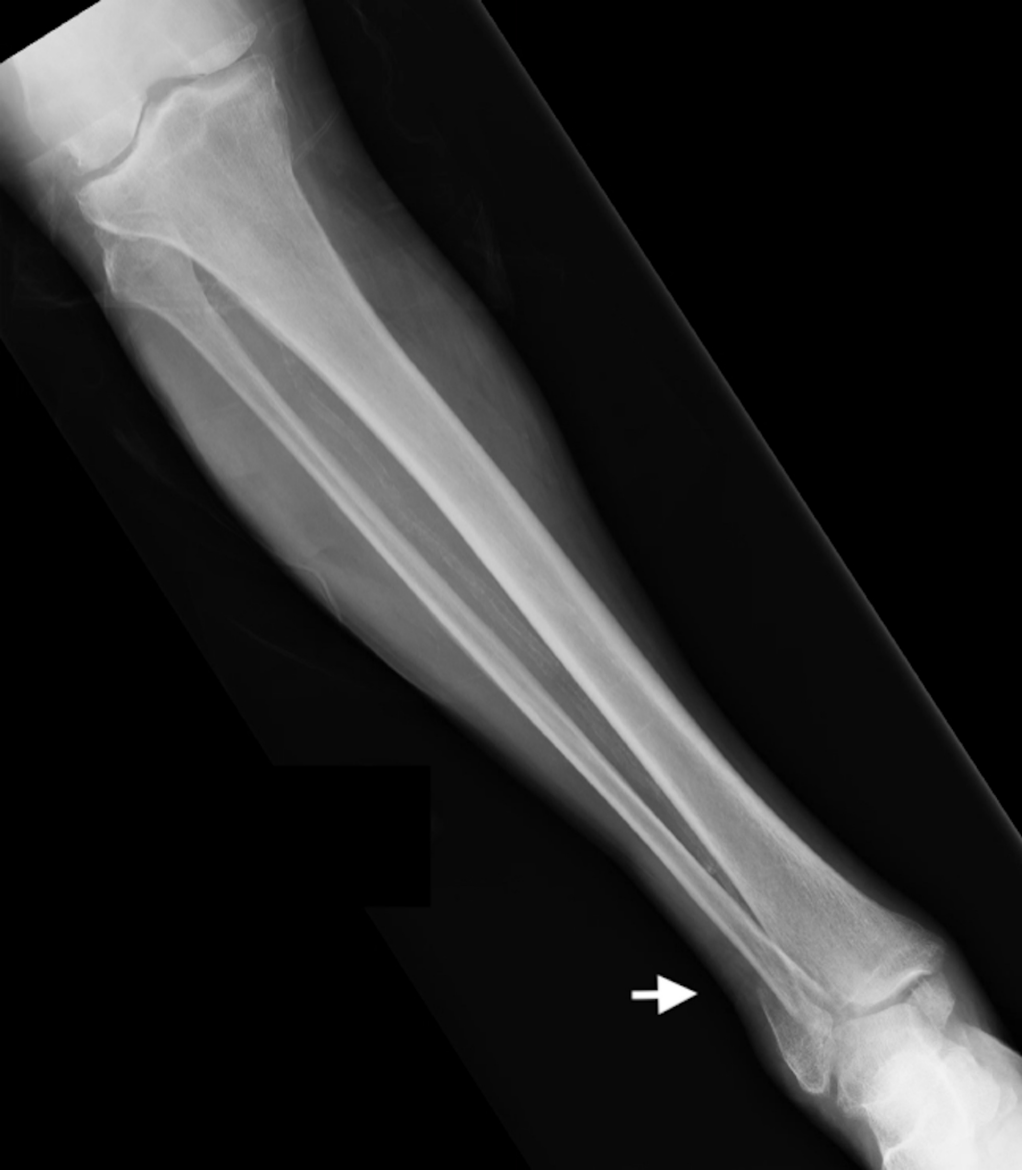
X-ray of right leg showing distal fibular fracture (arrow)

**Figure 2:**
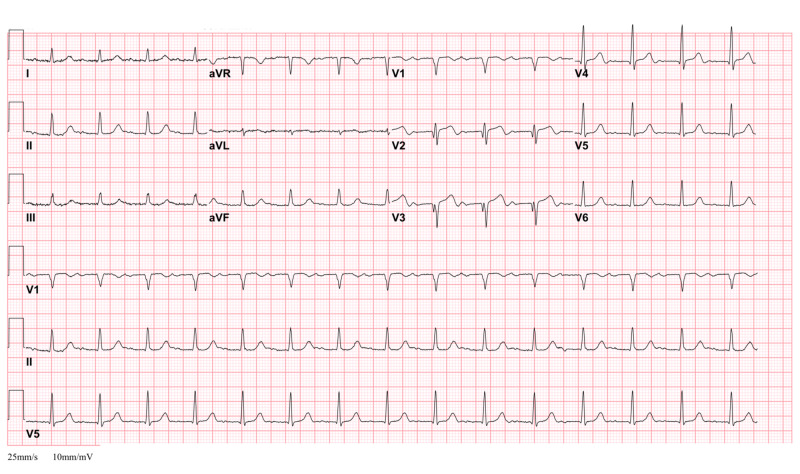
An electrocardiogram showing biphasic T wave in leads V2 and V3

A 76-year-old male patient with a past medical history of alcohol abuse presented to the emergency room (ER) with a right ankle pain that started after he sustained a mechanical fall. He denied chest pain, dizziness, lightheadedness, or palpitations. An X-ray of the right leg showed a right fibular fracture (Figure 1). The patient was admitted to our hospital for medical optimization prior to open reduction internal fixation (ORIF) surgery. An ECG done in the ER showed biphasic T waves in leads V2 and V3 (Figure 2), raising the concern for Wellens' syndrome. Initial troponin T levels were elevated to 0.9 ng/ml (normal reference range at our facility is less than 0.010 ng/ml). Subsequent workup showed an increase in troponin T levels to 1.6 ng/ml. 

**Figure 3:**
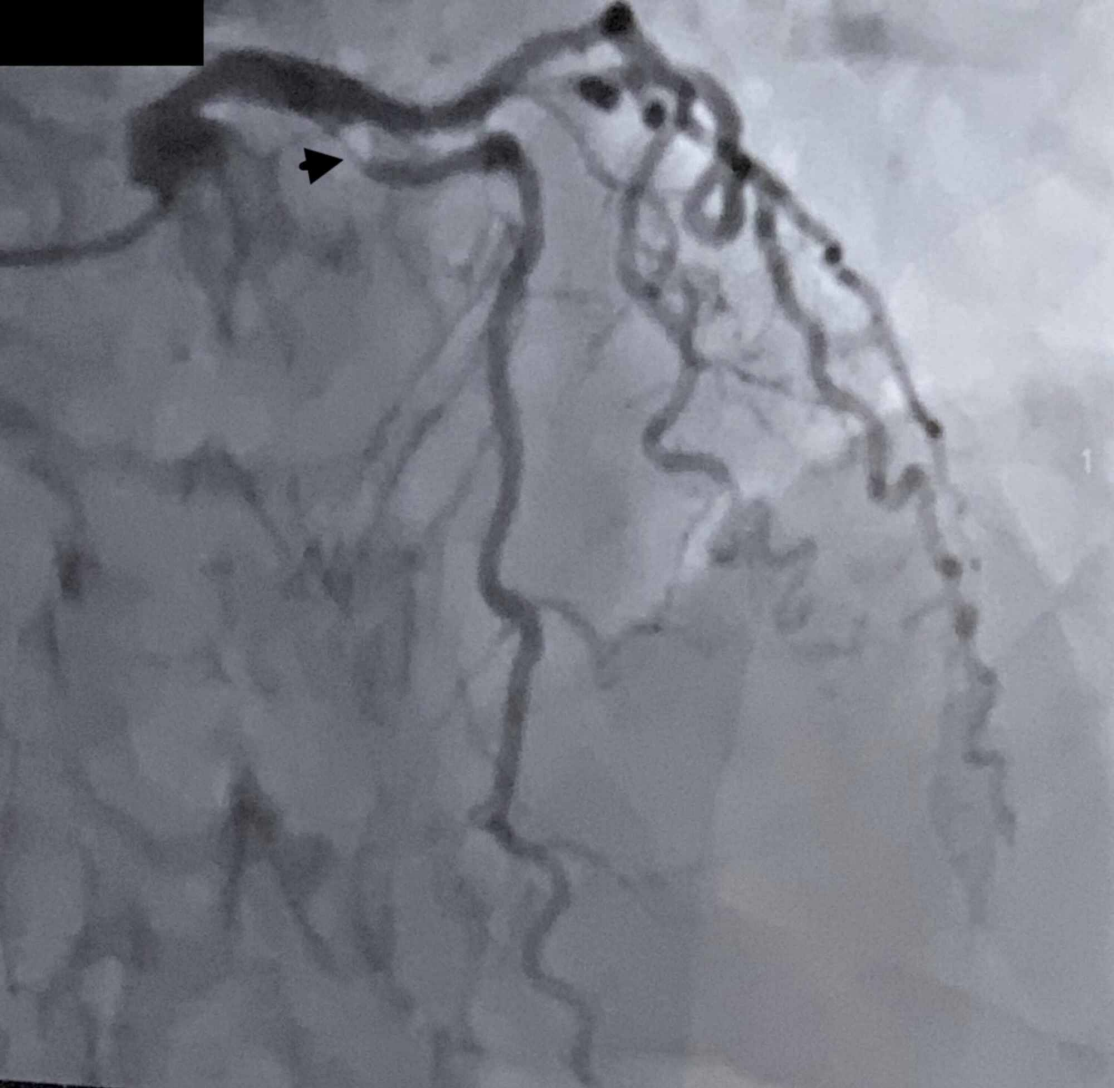
Coronary angiography showing 90% stenosis of the proximal left anterior descending artery (arrowhead)

The patient received aspirin, Plavix, and heparin. He was urgently transferred to the Cath Lab unit. Coronary angiography revealed severe and heavily calcified tortuous three-vessel coronary artery disease (CAD), including 90% stenosis of the proximal LAD artery (Figure 3), 75% stenosis of the first obtuse marginal branch of the left circumflex (LCX) artery, 80% stenosis of the second obtuse marginal branch of LCX artery, and 70% stenosis of right coronary artery (RCA). No intervention was done during cardiac catheterization. The patient had a coronary artery bypass grafting (CABG) surgery the next day using the left greater saphenous vein. After an ICU stay lasting 20 days, he underwent ORIF surgery of right fibula and was transferred to a regular floor. He was ultimately transferred to a rehab facility.

## Discussion

Wellens’ syndrome also known as ‘LAD coronary T-wave syndrome’ refers to the unique pattern of T-wave ECG changes that are associated with critical stenosis of the proximal LAD artery [1]. Recognition of this ECG abnormality is important as this syndrome represents a preinfarction stage of CAD that can progress to an anterior wall MI. Although the exact mechanism behind this ECG pattern is unknown, it is proposed that it is secondary to alleviation of proximal LAD artery spasm that leads to reperfusion of the ischemic myocardium [3]. Evolution to an anterior wall MI is usually rapid, with a mean time of 8.5 days from the onset of Wellens’ syndrome to infarction, which can result in ventricular dysfunction, putting the patient at risk for congestive heart failure [2]. 

Two ECG patterns of T-wave abnormalities are usually seen in Wellens’ syndrome. The more common pattern presents with a deeply inverted T wave in ECG leads V1 and V2; this pattern is called type B and comprises 75% of cases [1,3]. Less commonly, T wave can be biphasic in leads V2 and V3, with initial positivity and terminal negativity as seen on our patient’s ECG (Figure 2). This pattern is called type A and comprises 25% of cases.

The diagnosis of Wellens’ syndrome can be established when biphasic or deeply inverted T waves are found in leads V2 and V3 without significant ST elevation, along with normal precordial R-wave progression in the setting of recent anginal pain, and normal or slightly elevated troponin levels [1,3]. These diagnostic criteria have a positive predictive value of approximately 86% for Wellens’ syndrome [3]. 

The unique ECG patterns associated with Wellens’ syndrome are usually observed when the patient is pain-free [3]. Our patient had presented to ER for an ankle fracture; ECG on admission revealed biphasic T waves in V2 and V3 with an elevation of troponin T. Subsequent workup showed an uptrending troponin T levels while the patient remained chest pain-free throughout his course of illness. Despite the patient not having any chest pain, the characteristic ECG finding of Wellens’ syndrome along with the elevation of cardiac enzymes led to an urgent coronary angiography that revealed three-vessel CAD. Failing to recognize Wellens’ syndrome T-wave changes on ECG may lead to unnecessary and risky workup. Performing a stress test that includes pharmacological provocative agents puts the patient at risk of provoking a large anterior wall MI [4]. It is thus important to recognize Wellens’ syndrome on ECG and consult appropriate services to prevent fatal consequences. 

Even with ideal medical management that typically includes aspirin, Plavix, and heparin, Wellens’ syndrome ultimately progresses to an acute anterior wall MI, as approximately 75% of patients with Wellens’ syndrome who receive only medical management without undergoing surgical revascularization (like CABG or angioplasty) develop extensive anterior wall MI within weeks [1,2]. Thus, the definitive management for Wellens’ syndrome is urgent angiography and revascularization [2].

## Conclusions

Wellens’ syndrome presents with a unique pattern of T-wave changes on ECG that are specific for proximal LAD artery stenosis. It is important to recognize these findings on ECG because they can precede an anterior wall MI even without presenting with chest pain. Surgical revascularization of coronary vessels is the mainstay of treatment as medical management alone puts the patients at risk of developing an MI. 
